# Altruism, Solidarity and Responsibility from a Committed Sociology: Contributions to Society

**DOI:** 10.1007/s12108-021-09504-1

**Published:** 2021-08-06

**Authors:** Estrella Gualda

**Affiliations:** 1 ESEIS/COIDESO [ESEIS, Social Studies and Social Intervention Research Centre; COIDESO, Centro de Investigación en Pensamiento Contemporáneo e Innovación para el Desarrollo Social], Universidad de Huelva, Huelva, Spain; 2 Facultad de Trabajo Social, Universidad de Huelva, Campus El Carmen. Avda. Tres de Marzo, 21071 Huelva, Spain

**Keywords:** Altruism, Solidarity, Responsability, Common good, Refugees, COVID-19

## Abstract

A careful look at the international development of Sociology highlights the centrality that the study of social problems and the approach to possible solutions to them have had in the history of this discipline, not infrequently for the sake of better social integration, stability, development, social change or even modernity. Recent approaches suggest shifting this focus of attention, arguing about the deficit in sociological research and practice concerning theor etical frameworks that pay attention to the positive aspects. This text reflects on the contributions that altruism, solidarity, and collective responsibility can have to improve the quality of life in contemporary societies and face humanitarian emergencies with a certain degree of success. For instance, the so-called refugee crisis or the current COVID-19 pandemic poses significant challenges for societies. This article also explores briefly new roles of data science in connection with responsibility and altruism. The text invites us to revisit sociology, thinking about the lights more than the shadows.

In her recent book, Emiliana Mangone (2020) has reviewed the history of Sociological theory to explain how Sociology has approached the egoism and altruism dichotomy. She has proposed ways to overcome this dichotomy, drawing some lines that lead towards reinforcing approaches that promote a committed humanistic Sociology close to some developed notions of altruism in the Social Sciences.

Based on some of her ideas, this article presents some reflections on how some connections can be found between sociological theorizing regarding altruism and current advances concerning approaches to the Common Good, prosocial behavior, and the renewal of Evolutionary Sociology in Social Sciences. Similarly, from a perspective that attempts to reflect on the certain naivety or lack of viability perceived in approaches such as Sorokin’s creative altruism, we refer to two current examples: the so-called “refugee crisis in Europe” and the COVID-19 pandemic. Through these examples, we will try to make visible some of the contributions that the approaches mentioned above can make to Sociology and Society, but also some important limitations.

## Sociology, Altruism and Committed Social Science

Although it has not been in the foreground in international sociological reflection throughout the development of Sociology, the question of altruism has a broad trajectory that connects with the official origin of this discipline (Jeffries & others, 2006). The use of the term ‘altruism’ is attributed to Comte, associating it with actions that benefit others who are different from the individual, opposing a classic idea of ​​‘egoism’, which focuses on the excessive emphasis on self or self-interest. In Comte’s classic book on *A Discourse on the Positive Spirit*, altruism is associated with the positive spirit (as a positivism scientific thinking), as opposed to the egoism that he attributed to theological and metaphysical thought: “the positive spirit as the only one capable, by its nature, of directly developing the social sense, the first necessary basis of all healthy moral”… “For the positive spirit, man does not properly exist; only Humanity can exist” (Comte, 1982: 128, 130–131).

In an easy reading, altruism and egoism can seem two sides of the same coin: altruism, usually associated with generous social actions that are offered selflessly to others, compared to egoism, which sometimes leads to an excess of utilitarianism, sometimes guided by principles of survival that forget the benefit of everyone. In other interpretations, altruism and egoism may represent different nuances. Ferrater (1994: 129–130) recalls two ideas that are associated with the origin of altruism. The first is that when altruism serves the community’s interests, it responds to its interests in such a way that “to be an altruist is to be a sui generis egoist” (own translation). The second idea states that utilitarianism is not the basis of altruism, but the opposite: altruism connects with social impulses rather than with individual ones, essential in the human being.

In Comte’s vision (1982) and his exaltation of the positive or scientific spirit, in front of previous forms of thought, the altruistic attempt to achieve the public good “will become the source of personal happiness” (p. 131), as something which inevitably derives from his deductive scheme. Perhaps an important nuance is pointed out by Mangone (2020) in her recent book, *Beyond the Dichotomy Between Altruism and Egoism. Society, Relationship, and Responsibility*, when she states that Comte theorized that “human altruism is a natural instinct similar to egoism. They differ particularly for one aspect, the latter tending to the conservation of the individual, while altruism is oriented to the conservation of the species sometimes playing a major role in the maintenance and social development of mankind” (Mangone, 2020:87).

However, whether altruism is a kind of “group egoism” or a genuine social impulse, from the perspective of social action and its effects on society, beyond the moral principles or underlying interests that sustain them, it seems that an action designed to benefit the others, the community, could potentially have more valuable effects globally than an action aimed at an individual benefit. In times of historical crisis, as is the case currently posed by the COVID-19 pandemic, altruist actions could be decisive for the future of Humanity.

With more or fewer roots in these ideas, throughout the sociological discipline, altruism or related concepts such as solidarity have been in one way or another present in classical authors of Sociology. Durkheim is perhaps an outstanding example. His writings on integration/disintegration and different types of solidarity in society, with altruism as a critical factor in understanding some of his types of suicide, where the degree of social integration becomes an element that prevents it (Durkheim, 1985). An in-depth review of the contributions to this question from the classics can be found in Mangone’s book (2020).

In recent years, scientific productivity has increased in this field of study, having suggested proposals to create a specialization in thematic axes related to altruism, solidarity, and social morality (Jeffrey et al., 2006), arguing that there has been a kind of rediscovery of altruism by the social sciences, which becomes an analytical construct of them (Mangone, 2020). On the one hand, classic ideas of sociology are revisited, and on the other, new fields of work or application are suggested. In other disciplines, such as economics or psychology, although altruism is not taken as the central axis, other conceptualizations that have relevance stand out, such as those related to the Common Good or the prosocial behavior (Ostrom, 1990; Ostrom et al., 1993; Ostrom, 2009; Ostrom, 2010; Felber, 2012; Gómez y Gómez-Alvárez, 2016; Schroeder & Graziano, 2015). The following pages will pay attention briefly to some connections of interest of these approaches.

However, the rediscovery of altruism is very uneven if we look at the development of international sociology. In some cases, Pitirim Sorokin, considered one of the predecessors and founding leaders of the specialization in altruism, morality, and social solidarity, with Tolstoy, Addams, and Gandhi (Nichols, 2014: 149), has been an inspiration for some contemporary sociologists. The influence of Sorokin, a Russian-born sociologist who serves as Professor of Sociology at Harvard and much of whose scientific production is still much unknown in part of the international sociology, was largely overshadowed by the predominant functionalism in the United States since the mid-twentieth century.

Nichols (1989) reminds us of Sorokin’s evolution as “a case analysis of the deviant career in sociology”, explaining how his work passed from sweet moments “from positive deviance, discovery and rise stage” (1924–1930) to his progressive stigmatization and long eclipse, so that, at the end of his career, there was a phase of “rediscovery, reconciliation and return” (1963–1968). At that time, sociology was more entrenched in science and academia.

Sorokin, in his proposal on *The Reconstruction of Humanity* (1958), from a framework where society, culture and personality are conceived in an interrelated way as the “indivisible sociocultural trinity” (1958: 91), sets out some lines for the regeneration of humanity. Compared to other approaches of that time, perhaps one of the main contributions of Sorokin’s analysis, seen with the eyes of more than 60 years later to his writings, is his commitment to altruism, as a necessary element to save or “Cure” -in his words- the humanity. This approach connects with his other writings, highlighting the crucial power of love in this reconstruction (Mangone & Dolgov, 2020). Sorokin (1958: 61) rightly states that: “No human group can survive without a minimum of altruistic conduct among its members”. Moreover, his diagnosis that the actions undertaken at that time in the world were not going to lead to peace was not wrong. Wars and other calamities continue in the world. Another question, in a book that is dedicated to Gandhi and whose prologue recalls: “Bleeding from war wounds and frightened by the atomic Frankensteins of destruction, humanity is desperately looking for a way out of deathtrap” (p. 8), is perhaps the extreme confidence that Sorokin shows in altruism and love, or the viability of love in a world context that is, unfortunately, to a great extent exceptionally cruel at times, and very utilitarian at other times, frequently reminding us to Hobbes (2005), with his classic expression of selfishness in Leviathan (“man is a wolf to man”).

However, it is essential to remember that Sorokin defines different types of altruism and argues that altruism occurs with different intensities in Society. “Genuine altruism,” from his perspective, is pure altruism and is characterized by its non-utilitarian motivation. Moreover, it is wise and creative altruism, both objectively and subjectively, devoid of harm to others, as the acts are motivated only by continuous and lasting love. In Sorokin’s vision, the maximum degree of altruism would be inseparable from creativity, compared to other types of behavior (Sorokin, 1958: 62–67). Pure or genuine altruism, for some authors, cannot exist -or is practically impossible- (Spencer, 1873), and even it is not possible to be scientifically measured (Bykov, 2017). In Spencer’s words: “So that, pure altruism in a society implies a nature which makes pure altruism impossible, from the absence of those towards whom it may be exercised!” (1873:570).

Nowadays, it is difficult to think about the viability of love or altruism when looking at the daily problems surrounding us. However, on the international scene, influential institutions have been putting forward proposals along these lines, although they are not very successful if we remember the continuous armed conflicts, corruption, situations of poverty or other problems that international policies cannot solve. A significant example is provided by the international declaration of the United Nations in 2000 on the Millennium Development Goals that finally were not achieved (United Nations Development Programme, 2021). Currently, it is the Sustainable Development Goals, approved in 2015, that continue this line of work through the United Nations Development Program, with new challenges because of COVID-19 (United Nations, Human Rights Office of the High Commissioner, 2021; United Nations, 2020; United Nations Development Program, 2021).

Although, perhaps, one can generically agree with Sorokin’s view when he stated that: “At the present juncture of human history, a notable increase of an unselfish, creative love (goodness) in the superorganic world is the paramount need of humanity” (Sorokin, 1960, p. 184), a look at history and the current situation requires asking ourselves about the viability of creative altruism to solve Humanity’s problems, or if -better- other instruments of international policy would be equally necessary to be promoted (as, for instance, the above mentioned, and many others). In Mangone’s recent proposal (2020: 196), it is stated that although “it is not possible to imagine a world without selfish relationships,” ... “it is possible to imagine a world in which the negative consequences of these relationships are reduced to a minimum”. The assignment for Social Sciences and Sociology is, perhaps, to follow a path framed in the obligation to act from an “ethics of responsibility” (p. 187) and from a “Committed humanistic sociology” (Mangone & Dolgov, 2020, Mangone, 2020). The challenge that opens here would be to begin to draw concrete lines of work that make it possible, in a realistic way, to reach these goals.

## Theoretical Frameworks that Address Altruism and Solidarity Advantages to Benefit Individuals, Communities, and Societies

### Prosocial Behavior

In addition to classical approaches or authors such as those mentioned above, the Social Sciences have been incorporating theoretical approaches that provide a certain degree of optimism or possibilities for the future, identifying elements prone to generosity rather than selfishness in people, communities, or societies. An example of this is the approach that highlights prosocial behavior that seeks to know why there are altruistic people whose acts favor others more than oneself (Schroeder & Graziano, 2015). Knowing the origin and causes of altruism can help to promote this generous and beneficial conduct for humanity. With a more psychological approach and focused on prosocial behavior, understood as the antonym of antisocial (in Batson, 1987), in recent years attention has been devoted to improving our understanding of why people act to benefit or help others in different social instances. That is the case of the contributions in the *Oxford Handbook of Prosocial Behavior*, edited by Schroeder and Graziano (2015), which directly reminds us of contributions such as Sorokin’s in Sociology. Altruism broadly aligns equally with prosocial behaviors such as donating, sharing, cooperating, or helping. Wittek and Bekkers (2015) explain that prosocial behavior entails costs for the self and benefits for others. However, they clarify that although prosocial behavior is purely behavioral, altruism has motivational and behavioral components. In this sense, one can remember with Bykov (2017) different approaches in the study of altruism (as motivation, in psychology; behavior in evolutionary studies, or other approaches more linked to normative or structural components, as in sociology).

Prosocial behavior, it is argued, is key to achieving the well-being of human groups. Recently, the development of this idea is internationally influenced by evolutionary science, promoting social change (see, for example, in Prosocial World, 2021, https://www.prosocial.world/). Furthermore, this approach enhances the importance of cooperation and collaboration of social groups in different areas (Biglan, 2015, Prosocial Word, 2021). Clark et al. (2015) highlight that prosocial behavior is defined as the attempt by one person to promote well-being or prevent well-being from deteriorating. To achieve this end, they emphasize that the relational context is key to shaping relationships. The relational context of the interaction is also crucial because it defines the rules and norms that guide behavior. In this way, people act differently depending on the context in which they find themselves.

### Common Goods

On the other hand, while some approaches have focused their attention on altruism or the orientation towards benefiting others through prosocial perspectives, other lines of research has also highlighted how, at the community level, a common good approach in the management of common property assets provide social capital and advantages with social self-organization, producing positive effects for society.

In recent years, one prominent approach is focused on the common goods, based on the works of Ostrom and other authors (Ostrom, 1990; Ostrom et al., 1993; Ostrom, 2009; Ostrom, 2010; Felber, 2012), who highlight the importance of aspects of auto-organization for the improvement of the survival of some communities. In this case, which has macroeconomic and social dimensions, in connection with micro and meso elements, work for the common good represents in a certain way a type of organization that is committed to present and future survival. Cooperation in sharing resources from the shared pool is seen as the key here for the survival of the communities. Elenor Ostrom documents international examples of the advantages of “governing the commons” in places as diverse as Kenya, Guatemala, Nepal, Turkey, and Los Angeles. An essential element in this approach, which connects to some dimensions of altruism, is the argument that the commons can be governed sustainably and equitably in a community. The idea of ​​equity or the proposal that ‘common goods’ are adapted to local needs represents a connection with the idea of altruism as it goes beyond self-interest trying to solve community problems and personal necessities.

### Evolutionary Sociology

In another line of thought, the new Evolutionary Sociology, it is found that some authors reincorporate in sociology, parallel to other advances in other scientific fields, the look towards biology. Proposals have been made for the theoretical reconstruction of the sociological discipline from this line of evolutionary sociology. For example, Schutt and Turner (2019) and Turner et al. (2020) suggest that some paradigms from the past may be helpful to increase our understanding of human beings. There is a reformulation of these approaches, arguing that sociology has the opportunity to develop its own evolutionary focus, an approach to biology, as has been done in economics or psychology, for example. In this context, the idea of ​​natural selection persists but is reconceived as “multi-level selection.” Multi-level selection is a central aspect in the new evolutionary sociology, with claims to examine the relationships between biological and sociocultural elements.

Hopcroft (2016), for example, refers to the great challenge of evolutionary sociology and biosociology, areas in which it is sought to examine the interaction of environmental and social factors with biological ones. She argues that social behavior can be explained by considering both cultural and biological aspects since they are not exclusive, insofar as human culture results from our biological nature (Hopcroft, 2016). Along with other authors, she argued that the founders of sociology did not deny the role of biology or the importance of evolution. Nevertheless, incorporating the social Darwinian approach and sociobiology in the XX century -with ethnocentric, racist, fascist, or sexist purposes-, made the integration of sociology and biology difficult in the past (Hopcroft, 2016). The evolutionary sociology approach argues that the advancement of sociology as a social science involves reconnecting sociology with biology and the rest of life sciences after having learned history lessons.

This line of work, which seeks the return of evolutionary theory to sociology, is not without controversies (Ribeiro, 2009). For instance, the divergences between the community of sociobiologists and those aligned to evolutionary sociology. While the first come to build a second theory of social Darwinism, with great inspiration in approaches such as Wilson’s sociobiology (2000), the second proposes evolutionary sociology trying not to renounce biology but to develop an approach with its sociological entity.

In discussions about the new evolutionary sociology or even biosociology, the question arises of how altruism is born and its role. That is, what explains the existence of altruistic behaviors between individuals who are not genetic relatives.

Apart from the doubts above exposed regarding whether Sorokin’s genuine altruism may exist or not, perhaps an element of greater utility for sociology is considering the complexity of understanding how altruism emerges, or even if we are -as human beings- more selfish or altruistic. However, most important is for us to know how altruism and solidarity can be implemented for societal and community benefits with the support of institutions. In this sense, more than elements of a biological nature linked to evolution (or if you like, genetics), or behavioral (psychological), they are, in our view, the organizational and institutional aspects -understood in a broad sense- what truly matters to promote welfare in societies. On the other hand, ethical and moral elements are unavoidable at working with altruistic or solidarity approaches. They are needed if it is promoted a sociology committed to the well-being of people and the sustainability of communities.

Moreover, together with acting altruistically and supportively, it is necessary to draw a horizon of action based on respect for human rights or sustainability, for example, which goes beyond biological dimensions. In any case, independently if we consider altruistic notions, prosocial behaviors, or a focus aimed at preserving and enhancing common goods for the benefit of the community, these proposals have common elements. We refer basically to their trust in individual or organizational possibilities to contribute to the positive development of humanity.

Nevertheless, the lesson of history, or a simple look at contemporary reality, forces us to consider these approaches differently to a panacea due to the difficulties, which sometimes means putting altruism or solidarity into practice. A non-naive and non-deterministic perspective in the social, cultural, or biological spheres, seems equally essential given the abundant international experience regarding the difficulties of achieving equality, populations’ well-being, or the eradication of violence itself. The revitalization of evolutionary sociology approaches, or even sociobiology, beyond our biological component may be undeniable, seems to us less valuable to understand the functioning of social and cultural processes embedded in social structures, some of which consolidated throughout history.

On the other hand, as we will expose through the following examples (on the “refugee crisis” and the COVID-19 pandemic), the difficulties and complexities (political, social, economic, or cultural) involved in achieving solutions force the institutions to take a position and develop actions far from biological processes. Actions much closer to establish parameters where the defense of freedom, human rights, or the development of sustainable goals are decisive. In this framework, a committed sociology, where values ​​such as equity or solidarity are fundamental or altruistic behaviors become realities beyond biological notions, is critical.

In this sense, in the following pages, we try, very briefly, to refer precisely to two examples of international relevance that require caution regarding the scope or possibilities of short-term success of proposals based exclusively on some genuine altruism or prosocial behavior, idealized or naive.

### Institutional Responsibility, Solidarity, and Altruism: On the Refugee Crisis

The recent humanitarian so-called “refugee crisis” in Europe, as a result of the fact that more than one million immigrants crossed the Mediterranean in 2015, escaping from situations such as armed conflicts or persecutions, and frequently risking their lives (BBC, 2016; European Parliament, 2021), is a significant example of international scope, and with great impact on some European societies in recent years. This crisis allows us to reflect briefly on how various aspects of institutional responsibility are linked to altruism and social solidarity. On the other hand, the crisis itself and its development are also evidence of how far we are still from applying principles of creative altruism and love developed in Sorokin. The situation of refugees in the world, evaluated realistically, is an example of how immense inequalities are still to be resolved. Although international solidarity actions are indeed being deployed, the social, political and economic structures behind their situation (in their countries of origin or the places of destination) require significant changes. As a crisis of refugee protection mechanisms (Pries, 2019), this crisis is a clear example of the relevance of coordinated international policy actions. Guterres, the head of the UN refugee agency, stated that “It (the EU) now has no other choice but to mobilize full force around this crisis. The only way to solve this problem is for the Union and all member states to implement a common strategy, based on responsibility, solidarity and trust,” he said (Guterres, in Clayton, 2015, para. 7).

On the other hand, Twitter is an excellent example to observe some evidence of international solidarity with refugees looking at expressions of solidarity that take place on social networks and develop at both the institutional and personal level. Different campaigns, which have been held annually, are an example of this. Along with generic hashtags such as #DíaMundialdelosRefugiados or #WorldRefugeeDay, other common expressions of solidarity and altruism show more clearly the solidarity component. That is the case of #withrefugees from a UNHCR campaign (Rebollo, 2021), in which the speech focuses not only on the vindication of rights but on aspects where solidarity and humanity connect: “Cities stand #WithRefugees Over 250 cities worldwide have signed a statement supporting refugees and are asking more to join them” (UNHCR [The UN Refugee Agency], 2021).

Apart from this type of discourses supporting refugees [“We stand with Refugees”], critical citizens appeal to the institutions’ responsibility to promote solidarity policies. Furthermore, when this is not the case, and public institutions do not provide a solution to the human drama, responsibility is attributed to them, sometimes metaphorically. One example of this is symbolically represented by some hashtags such as #UEmata, #UErfanos or #vergUEnza -in Spanish- (Gualda and Rebollo, 2016). To understand what is symbolized in these campaigns, it must be explained that the EU is equivalent to the European Commission or the European Union. #UEmata hashtag suggests that the European Union does nothing to solve the refugee crisis. Concretely, by “UEmata” in Spanish, it is suggested that “The European Commission is responsible for the death of refugees”. Another case is “UErfanos”, which refers to the word “orphans” (with the same sound in Spanish). There is a reference here to the deaths in the Mediterranean. By #vergUEnza, on the other hand, emotions are appealed ("vergüenza" is equivalent to the English word "shame").

In addition to the feelings of shame due to the scarcity of solutions provided to this humanitarian drama, various NGOs launched campaigns in which a clear responsibility was attributed to European institutions. Responsibility is attributed based on the belief in a human rights framework deeply rooted in the mentality of many Europeans, where, confronted with utilitarian approaches, there is a philosophy of solidarity, although not always consistent with the dramatic events that some crises reveal.

Jeffrey and others (2006) suggest, as a challenge for the sociology that shifts its attention from problems to the *advantageous* aspects of society and its social organizations, the importance of defining the good and considering studying it critically. In this sense, it is argued that a part of public sociology should be made up of dialogues about the good and the positive. An example is the study of human rights and the conditions for their realization. Another example would be the study of altruism and solidarity as a recognized field of expertise.

Returning to the discourse on refugees, the humanitarian discourse that is constructed is complex. Sometimes it appeals to the morale of citizens. Other times is focused on the imagination of what is supposed to be desirable or not in each society. Thus, together with a critical vision of the institutions, it appeals to emotions and compassion, trying to humanize refugees and nurture feelings of empathy and solidarity (Rebollo, 2021). However, sometimes humanism becomes instrumental when invoking compassion is linked to campaigns for obtaining funds to intervene in vulnerable groups. Invoking emotions and morality to remind citizens of their social values as a strategy makes us forget that international law principles should protect refugees from the drama recently experienced in the Mediterranean in Europe. The lack of security and tremendous vulnerability on their trip is also portrayed through campaigns such as #SafePassage [in Spanish: #PasajeSeguro, # VíasSeguras], in which NGOs such as the Spanish Commission for Refugee Aid [CEAR], International Amnesty or Oxfam Intermón, among others, participate.

Concerning this humanitarian crisis, solidarity, responsibility and the common good are proposed in the same narrative by some citizens, which provides another vision, optimistic about the possibility of intervening, but with a perhaps less emotional focus. The following tweet is an example:

Baracaldo, A.M. [@AnaBaracaldo]. (2019, June 19). There are over 70 million people #displaced by war, persecution and conflict. It is time for solidarity, for shared responsibility, for a common Good [tweet]. Twitter https://www.unhcr.org/cgi-bin/texis/vtx/refdaily?pass=52fc6fbd5&id=5d09daab3… #withrefugees #solidarity. https://twitter.com/AnaBaracaldo/status/1141367184838602755

However, we can find evidence of other narratives that emerged during this crisis in support of the refugees. They represent different existing visions on how to handle the refugee crisis. In this case, returning to messages published on social networks regarding how citizens are appealed to, some statements refer altruism and compassion (for example, in Trudeau, as First Ministry of Canada in 2016). Other messages suggest the relevance of human rights. We have frequently found this diversity of messages suggesting different strategies for intervention regarding refugees in our research (Gualda and Rebollo, 2016):

Trudeau, J. [@JustinTrudeau]. (2016, 20 de junio). “On #WorldRefugeeDay, we recommit to helping the most vulnerable in the spirit of compassion & generosity. #WRD2016” [tweet]. Twitter.

https://twitter.com/JustinTrudeau/status/744895518917042176

Taim Shami, N. [@Nael_TaimShami]. (2016, 25 de junio). “We stand #WithRefugees. Their rights must be respected. Their dignity must be protected. #WorldRefugeeDay @UN_Women” [tweet]. Twitter. https://twitter.com/Nael_TaimShami/status/746678669356175360

### Altruism, Common Goods, Collective Responsibility and COVID-19

The COVID-19 pandemic, in which the world has been immersed for more than a year, poses new challenges to Humanity, which, although not new, suggest the need for progress in strategies and actions that minimize the negative effects of it. Some proposals for addressing the current pandemic come from complementary frameworks of action. For instance, the international human rights approach (United Nations, 2021). Also, the 17 sustainable development goals resulting from the United Nations approval in 2015, framed in the 2030 Agenda for Sustainable Development. Achieving these goals in the context of COVID-19 provides a framework for recovery. The United Nations (2020: 11) point out that they require outstanding political leadership and cooperation to combat COVID-19: “At the geopolitical level, this crisis cries out for leadership, solidarity, transparency, trust and cooperation. This is no time for self-interest, recrimination, censorship, obfuscation or politicization”.

On the other hand, there are also appeals to the “Common Good” to help solve the problems generated by the pandemic (Agazzi, 2020). Beyond religious or philosophical considerations about the Common Good that can be traced in theology, philosophy or, for example, in political science (Longley, 2020; Hussain, 2018), the severity of the pandemic has even produced petitions such as that vaccines are considered a Common Good for Humanity (Yunus et al., 2020; also at: https://vaccinecommongood.org/). Current approaches to the Common Good in economics, political science and even sociology (Ostrom, 1990; Longley, 2020; Felber, 2012; Perkiss & Moerman, 2020) recall, in situations such as the current one, the importance of what benefits all members of a community in contrast to individual benefits. In this sense, this type of approach is somewhat aligned with some of Sorokin’s ideas, as it goes beyond utilitarian approaches, although perhaps more operationally and concretely. They also remind us that, faced with the losses caused by any disaster (alluding to their work on calamities), individuals and their communities, as Mangone and Zyuzev (2020: 189) stated, always find the opportunity to adapt and grow.

Until now, the development of the pandemic at the international level has allowed us to observe different types of solidarity experiences at the international, national, regional and local levels, which have contributed to helping to resolve some social emergencies, albeit minimally. However, at the same time, negative experiences connecting with egoism are present. For instance, there is competition for vaccines and health material since the beginning of the pandemic. Also, some experiences of corruption in different countries emerged, giving priority to vaccination to some people over others with non-medical criteria. Also, authorities warned of how the pandemic has abounded in the stigmatization and discrimination of already disadvantaged groups. Asians, refugees, immigrants, Rome, women, Jews, and LGBTI people were recipients of expressions of hatred and arguments that blame them for the pandemic (European Commission against Racism and Intolerance, 2021).

On the other hand, if during the pandemic many countries used the rhetoric of a “warrior metaphor” [you have to fight both a pandemic and an infodemic] (Gualda, 2021: 268), which served many governments to urge their citizens to comply with the health guidelines, calls for responsibility, social discipline and solidarity were also frequent, which was symbolically represented in public communications through not only messages from #StayAtHome, but also with others related to unity and responsibility that different government bodies disseminated. In Spain, the following was recurrently visited: #EsteVirusLoParamosUnidos (#WeWillStopThisVirusTogether).

The revitalization of an anti-vaccine movement develops at the same time that these expressions of solidarity and unity for overcoming the pandemic. Likewise, the emergence of a denialist movement reluctant to admit the existence of COVID-19 and anti-masking groups become relevant. Even despite the high rates of infection and international mortality (John Hopkins University, 2021) and the drama that COVID-19 has caused in various countries. As examples, remember the news of graves in Brazil (León, 2020), corpses in Ecuador (Watson, 2020), the current high mortality in India (BBC, 2021), or even the high number of Americans who seem to resist the vaccination (Monmouth University Poll, 2021). These examples are a reminder that the international search of mechanisms for the solution of severe Humanity problems cannot depend on naïve approaches or individual hands.

### Future Avenues and the Need for Viable Proposals

Seen as a whole, due to the drama and mortality that the pandemic is causing (as a relevant example), it is not easy to imagine as viable, on the occasion of the discussion on the recent book by Mangone *Beyond the Dichotomy Between Altruism and Egoism* (2020), Sorokin’s noble proposal regarding creative altruism and the power of love (Sorokin, 1960). Apart from considering that we are very far from achieving these goals globally.

Ideas around creative altruism and the power of love are proposals on which Humanity and Sociology should undoubtedly reflect. Nevertheless, the line of arguments that we consider most productive for current sociology is trying to combine diagnosis with viable proposals. It means trying to understand and explain social complexity from approaches that triangulate different levels of analysis. Not naïve proposals of intervention, operative or achievable in the short or medium term. Without perhaps losing sight of a much more distant and less viable desirable horizon. Some theoretical lines that combine these levels of analysis with notions such as altruism are already pointed out in the recent book by Mangone (2020).

However, in light of recent experiences, other fields for Sociology can be suggested where the excessive centrality that the discipline has placed in highlighting problems or pathologies could be compensated. Along with ideas that revisit altruism or emphasize the Common Good, prosocial behavior, the achievement of human rights, or even Sustainable Development objectives, sociology can also make significant contributions in other promising current fields. Of particular interest is if the orientation towards social problems is enriched by focusing on solutions and good practices to overcome them.

The emerging field of Big Data [area of work devoted to collect, store and analyse large datasets] has been very useful in some areas that highlight the economic value or strategic importance that big data can produce for companies (Jin et al., 2015; Del Vecchio et al., 2018; Bartosik-Purgat, 2018). From a mixed qualitative and quantitative approach, sociology could also enrich this area of knowledge, reinforcing its look to focus on what produces higher social value. That could be a way to contributing to a new computational Sociology (Edelmann et al., 2020) that deploys sociological imagination in this field (Evans & Foster, 2019). From this approach, it is possible to provide new analysis and propose solutions to be implemented in emergencies and humanitarian catastrophes and armed conflicts, violence or terrorism, and even in everyday life.

We could wonder how from mixed sociology linked to data science, experiences of solidarity and altruism could be collected and analyzed (from the local to the international level). One goal of this task is to provide higher visibility to altruistic and solidary solutions and experiences, linked to the Common Good, the sustainable development goals, or the human rights that can serve our collective learning. In this sense, together with repositories of natural disasters, terrorist attacks or armed conflicts, a promising line for research, among others, could be the systematic orientation towards collecting and analysing different international experiences of altruism and solidarity that collective intelligence has provided to Humanity.
